# Surfactant in a Polyol–CO_2_ Mixture: Insights from a Classical Density Functional Theory Study

**DOI:** 10.1021/acs.langmuir.2c02913

**Published:** 2022-12-16

**Authors:** Sriteja Mantha, Huikuan Chao, Andrew S. Ylitalo, Thomas C. Fitzgibbons, Weijun Zhou, Valeriy V. Ginzburg, Zhen-Gang Wang

**Affiliations:** † Division of Chemistry and Chemical Engineering, California Institute of Technology, Pasadena, California 91125, United States; ‡ Dow, Inc., Midland, Michigan 48667, United States; § Dow, Inc., Lake Jackson, Texas 77566, United States; ∥ Dow, Inc., Midland, Michigan 48667, United States; ⊥ Michigan State University, East Lansing, Michigan 48910, United States

## Abstract

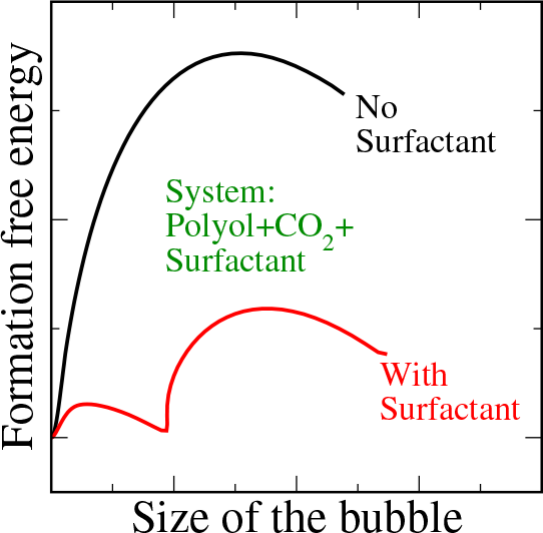

Silicone–polyether (SPE) surfactants, made of a polydimethyl-siloxane (PDMS) backbone and polyether branches, are commonly used as additives in the production of polymeric foams with improved properties. A key step in the production of polymeric foams is the nucleation of gas bubbles in the polymer matrix upon supersaturation of dissolved gas. However, the role of SPE surfactants in the nucleation of gas bubbles is not well understood. In this study, we use classical density functional theory to investigate the effect of an SPE surfactant on the nucleation of CO_2_ bubbles in a polyol foam formulation. We find that the addition of an SPE surfactant leads to a ∼3-fold decrease in the polyol–CO_2_ interfacial tension at the surfactant’s critical micelle concentration. Additionally, the surfactant is found to reduce the free energy barrier and affect the minimum free energy pathway (MFEP) associated with CO_2_ bubble nucleation. In the absence of a surfactant, a CO_2_-rich bubble nucleates from a homogeneous CO_2_-supersaturated polyol solution by following an MFEP characterized by a single nucleation barrier. Adding a surfactant results in a two-step nucleation process with reduced free energy barriers. The first barrier corresponds to the formation of a spherical aggregate with a liquid-like CO_2_ core. This spherical aggregate then grows into a CO_2_-rich bubble (spherical aggregate with a vapor-like CO_2_ core) of a critical size representing the second barrier. We hypothesize that the stronger affinity of CO_2_ for PDMS (than polyether) stabilizes the spherical aggregate with the liquid-like CO_2_ core, leading to a lower free energy barrier for CO_2_ bubble nucleation. Stabilization of such an aggregate during the early stages of the nucleation may lead to foams with more, smaller bubbles, which can improve their microstrustural features and insulating abilities.

## 1 Introduction

Polymer foams are lightweight materials with gaseous voids trapped in a polymer matrix.^1−7^ Their properties depend strongly on microscopic features such as the size, density, and connectivity of the gaseous voids in the material.^8,9^ The gaseous pores can be entirely separated from each other by the polymer matrix, leading to a foam with a closed-cell structure.^2,4,5,10^ Alternatively, the gaseous pores can be interconnected within the polymer matrix, forming an open-cell foam.^2,10^ The closed-cell foams are rigid and are good thermal insulators. Consequently, they have found applications as materials in the construction, refrigeration, and automotive industries. Open-cell foams, on the contrary, are soft and flexible. These foams are used as materials for sound insulation and cushions for furniture, among many other applications.

Microscopic features of a foam are significantly influenced by the foam production process.^3^ A standard procedure for producing foams^2,4^ involves generating bubbles and stabilizing them within a polymer matrix. Reactive foaming takes advantage of chemical reactions between the blending reactants for gas evolution and their nucleation within a dense polymer medium. On the contrary, in a physical foaming process, the polymer is first saturated with gas at a desired pressure. Then the system conditions are instantly changed to initiate nucleation of gas bubbles in the system. This process yields a metastable condition in which the system is supersaturated with gas in the polymer. Such a supersaturated system evolves through nucleation of gas bubbles and subsequent growth of thus nucleated bubbles.

The presence of gas bubbles in a liquid makes a foam an inherently unstable system.^11,12^ As a consequence, a foaming material ages over time. Drainage of liquid from the film between the bubbles,^13,14^ bubble coarsening,^15^ and bubble coalescence^16^ are three main processes that contribute to foam instability. Surfactants are commonly used to stabilize foams against aging.^12,17,18^ These molecules adsorb at the gas–liquid interface, improve interfacial properties, and constrain bubble coalescence and coarsening.^19−21^

Many studies in the literature have investigated the effect of a surfactant on the insulating properties and the mechanical strength of surfactant-stabilized foams.^22−30^ The most striking observation from these studies is that the addition of a surfactant yields a foam with a reduced cell size, an increased cell density, and an improved uniformity in cell size. Though the microstructural features of a foam are affected by both bubble nucleation and growth, Zhang et al. highlighted that they are more sensitive to the parameters governing bubble nucleation than those governing bubble growth.^31^ While the role of a surfactant in stabilizing bubbles to achieve these properties has been reported,^32^ its role in the nucleation of bubbles has not been reported, despite the high sensitivity of the nucleation barrier to interfacial tension.^33,34^ We attempt to bridge that gap through this study.

Classical nucleaton theory (CNT),^35^ classical density functional theory (cDFT),^36^ and molecular simulation techniques^37^ are commonly employed to investigate nucleation phenomena. CNT describes nucleation as formation of a new phase within a bulk phase. The formalism includes essential physics that governs the thermodynamic driving force for the formation of a new phase and the penalty for the formation of an interface between the new phase and the bulk phase. However, CNT uses equilibrium interfacial tension (IFT) and assumes a sharp interface. Such a theory is valid only near the coexistence. A semiempirical correction, called the Tolman correction,^38^ is often employed to model the radius-dependent interfacial tension. When nanoscale bubbles are being studied, such a modification is less accurate.^39^ There are also reports that invalidate the high free energy barriers predicted by CNT.^40^

Additionally, the theory does not include any molecule specific aspects of the system. Molecular simulations can alleviate some of the problems posed by CNT. However, they suffer from finite size effects that arise while simulating nucleation in a small system with a fixed number of particles.^41^ Though one can simulate a very large system^41^ or simulate by imposing a constant chemical potential,^42^ such simulations are computationally intensive.

cDFT^43,44^ coupled with the string method^45^ provides an appropriate framework for capturing the essential structure and thermodynamics associated with bubble nucleation. cDFT is a mean-field approach in which the free energy of the system is expressed as a function of spatially varying molecular densities. The density profile representing the equilibrium state of a system is determined by variational extremization of the free energy functional. If we have two such states, for example, state A being the homogeneous bulk and state B being a fully formed bubble of the prescribed size, a transition-state path-finding algorithm like the string method^45^ finds a minimum free energy path (MFEP) that connects states A and B. Though such an approach does not yield any dynamic information to go from state A to state B, it is still a very powerful technique for characterizing the MFEP and associated free energy barriers for the formation of a critical nucleus.

Xu et al.^46−49^ have successfully employed cDFT with the string method to characterize the barriers for the nucleation of CO_2_ bubbles in homopolymers like poly-methyl methacrylate (PMMA) and polystyrene (PS). In all of these studies, the authors have modeled their free energy functional for the cDFT based on the perturbed chain statistical associating fluid theory (PC-SAFT) equation of state (EoS).^50,51^ Because the PC-SAFT EoS and cDFT have been demonstrated to quantitatively describe the gas solubility and interfacial properties in CO_2_–PS and CO_2_–PMMA systems, we employ the same approach to model surfactants and study their effect on bubble nucleation in polymer foams.

We specifically investigate the effect of a silicone–polyether (SPE) surfactant on CO_2_ bubble nucleation in polyol. SPE surfactants are made of a polydimethylsiloxane (PDMS) backbone and polyether branches.^18,52,53^ While alkylethoxylate surfactants^19^ are ineffective in reducing the air–polyol interfacial tension, SPE surfactants are reported to significantly reduce the corresponding interfacial tension.^19^ This makes SPE surfactants indispensable as stabilizers in the production of ubiquitous polyol-based foams like polyurethanes. The addition of co-solvents is known to promote or inhibit the surface-active abilities and aggregation behavior of surfactants in solution.^54−57^ However, there have been no studies on how the presence of gas molecules would influence a surfactant’s activity. Understanding these properties and how they manifest in the nucleation of gas bubbles is particularly relevant to characterizing the role of surfactants in foam production.

Using the cDFT based on the PC-SAFT EoS approach described above, we characterize the interfacial properties and aggregation behavior of SPE surfactants in a mixture of polyol and CO_2_. Then, using the string method, we compute the MFEP associated with the nucleation of a CO_2_ bubble from a homogeneous mixture of CO_2_, polyol, and an SPE surfacant. Our main finding is that the SPE surfactant opens a low-energy barrier nucleation pathway. This has significant implications on the propensity for bubble nucleation and the resultant microstructural features of a foam.

The rest of the manuscript is organized as follows. We describe our models and cDFT approach in section 2. We report the results from our calculations and discuss them in section 3. We then conclude the article with an outlook on the path forward for developing foams with better physical properties.

## 2 Models and Methods

### 2.1 Molecular Model and the Helmholtz Free Energy Functional

We employ cDFT to model the CO_2_–polyol–surfactant ternary system. In our study, polyol is a homopolymer of poly(propylene oxide) (PPO) and the surfactant is a linear diblock copolymer with one block being PDMS and the other being PPO. Each of these molecule types is modeled as a tangentially connected chain of spherical beads. The Helmholtz free energy functional of such a system is then expressed as a sum of different perturbation contributions to the reference-state free energy functional.

To construct the cDFT, Xu et al. used weight-density functionals^58^ and extended the PC-SAFT EoS to model the free energy functional for the inhomogeneous system. For the systems of interest to this work, we follow the same procedure and write the Helmholtz free energy functional [*F*({ρ})] as
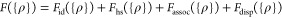
1where *F*_id_ is the ideal gas contribution, *F*_hs_ is the excluded volume contribution due to hard sphere repulsion, *F*_assoc_ is the association free energy due to the formation of a chain type molecule, and *F*_disp_ represents dispersion interactions between segments of different molecule types in the system. Each of these perturbation contributions to the free energy density is expressed as a function of the spatially varying segmental density ({ρ}) of the different components in the system.

In the PC-SAFT EoS, the properties of a given molecule of type *i* are specified by the pure component parameters *N*_*i*_, σ_*i*_, and ϵ_*i*_, which represent the number of segments per molecule, the size of the segment, and the strength of the interaction between segments of the same type, respectively. The interaction potential between any two segments of types *i* and *j* is described by
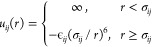
2where σ_*ij*_ = (σ_*i*_ + σ_*j*_)/2 and . *k*_*ij*_ is the binary interaction correction term that is used to account for any missing interactions between segments of types *i* and *j*. If ρ_*i*_(*r*^*N*_*i*_^) is the multidimensional density profile of molecule *i* with *N*_i_ segments, where *r*^*N*_*i*_^ = (*r*_1_, *r*_2_, ..., *r*_ζ_..., *r*_*N*_*i*__), the corresponding segmental density [ρ_*i*_(*r*)] can be expressed as
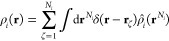
3The different contributions to the Helmholtz free energy, listed in eq 1, are expressed as a function of these densities defined in eq 3.

The ideal term (*F*_id_) of the Helmholtz free energy is known exactly:
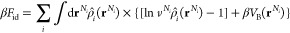
where *v* is a volume scale that has no thermodynamic consequence as it just shifts the chemical potential by a constant. *V*_B_ is the bonding potential that is used to enforce the chain connectivity between nearest-neighbor segments along the chain, and β = 1/*k*_B_*T*, where *k*_B_ is the Boltzmann constant and *T* the temperature. *V*_B_ is defined as
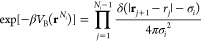
4

The excluded volume contribution (*F*_hs_), due to hard sphere repulsions, is modeled using the fundamental measure theory.^59,60^
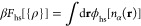
5with
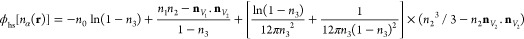
6where *n*_*j*_[{ρ}] = ∑_*i*_*n*_*ji*_ (*j* = 0, 1, 2, 3, *V*_1_, or *V*_2_) are the Rosenfeld weighted density functionals.^58^ These scalar and vector weighted density functionals are defined as
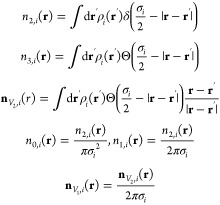
7In these equations, δ(**r**) is the Dirac delta function and Θ(**r**) is the Heaviside step function. The same weighted density functionals are used to describe other short-range interactions such as association and the local part of the dispersion interactions.

The thermodynamic perturbation theory of order 1 (TPT-1)^61−64^ is used to model the contributions to the free energy due to the association type interactions (*F*_assoc_). These interactions represent correlations within a molecule that arise due to the chain connectivity between the segments. If *i* = α, β, and γ index CO_2_, polyol, and surfactant segments, respectively, then the TPT-1 expression for *F*_assoc_ is given by
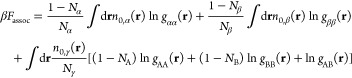
8where *N*_A_ and *N*_B_ are the number of segments in the A and B type blocks of the surfactant chain, respectively, and *N*_γ_ = *N*_A_ + *N*_B_ is the total number of segments per surfactant chain. Similarly, *n*_0,γ_(**r**) = *n*_0,A_(**r**) + *n*_0,B_(**r**), where *n*_0,*i*_(**r**) is the weighted density functional defined in eq 7. *g*_*ij*_(**r**) is the contact value of the correlation function between the segments of type *i* and *j* and is given by the expression in eq 9.
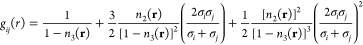
9

The contribution of dispersion (*F*_disp_) to the free energy has local and nonlocal components. The local term (*F*_disp–local_) is expressed as a perturbation to a chain-like reference fluid.^50,51,61,62,65,66^ This is obtained by directly extending the corresponding PC-SAFT EoS expression to the inhomogeneous system using the weight density functionals defined in eq 7. The resulting expression is shown in eq 10.
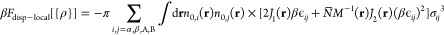
with
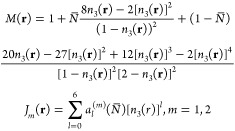
and the coefficients
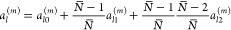
10where *N̅* = ∑_*i*_*N*_*i*_*x*_*i*_ and *x*_*i*_ is the mole fraction of molecule *i*. The constant coefficients, {*a*_*ln*_^(*m*)^|*m* = 1, 2; *l* = 0, 1, 2, ..., 6; *n* = 0, 1, 2}, are obtained by fitting the calculated binodal of the EoS with the experimental data for a great number of species. The values of these coefficients can be found in ref (51).

The *F*_disp–local_ term in eq 10 alone is not sufficient to describe the contributions due to dispersion interactions.^67^ A mean-field expression is included to account for any additional contributions due to spatial inhomogeneity.^68^ The corresponding nonlocal dispersion free energy term (*F*_disp–nonlocal_) is given by
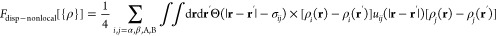
11

### 2.2 Euler–Lagrange Equations and the cDFT Numerical Procedure

In a cDFT approach, the equilibrium state of the system is determined by minimizing its grand potential (Ω[{ρ}]). The Helmholtz free energy functional (*F*[{ρ}]) defined in eq 1 is related to the grand potential of the system by
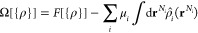
12where μ_*i*_ is the chemical potential of the *i*th molecule and ρ̂_*i*_(**r**^*N*_*i*_^) and ρ_*i*_(**r**) are the corresponding molecular and segmental density profiles, respectively, as defined in eq 3. When *F*^ex^[{ρ}] = *F*[{ρ}] – *F*_id_[{ρ}], extremizing the grand potential with respect to the molecular densities [ρ̂_*i*_(**r**^*N*_*i*_^)] results in the following Euler–Lagrange equations.
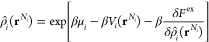
13

Using eq 3 and the corresponding relation for the functional derivative [i.e., ], eq 13 can be re-expressed in segmental densities [ρ_*i*_(**r**)] as
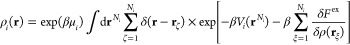
14

Equation 14 can be further simplified by introducing a recursive function
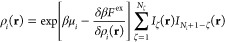
15where the recursive function *I*_ζ_(**r**) is given by
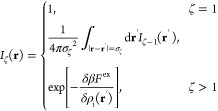
16

Equations 15 and 16 are the key equations for numerically computing the equilibrium density profiles of the different components in the system. In this work, we solve these cDFT equations in one-dimensional Cartesian coordinates and in spherical coordinates. The latter is used to study the properties of micellar aggregates and in the context of the string method to compute the MFEP for the nucleation of a spherical bubble.

The equations are solved in one-dimensional Cartesian coordinates to study the properties of the planar interface between the CO_2_-rich vapor and the polyol-rich liquid. To determine the spatial density profiles across the planar interface, we first compute the densities ({ρ_*i*_^v^, ρ_*i*_^l^}) of the different components (*i* = CO_2_, polyol, or surfactant) in the coexisting vapor (*v*) and liquid (*l*) bulk phases. If *f*({ρ}) is the Helmholtz free energy density of a bulk phase, then the corresponding pressure (*p*) is given by . At a given temperature, pressure, and surfactant concentration in the liquid phase, the densities in the coexisting bulk phases are obtained by searching for the condition of equality of chemical potential, i.e., μ_*i*_^v^({ρ^v^}) = μ_*i*_^l^({ρ^l^}), and the equality of pressure, i.e., *p*^v^ = *p*^l^ = *p*. These densities ({ρ_*i*_^v^, ρ_*i*_^l^}) are then used as Dirichlet boundary conditions [ρ_*i*_(0) = ρ_*i*_^v^; ρ_*i*_(l) = ρ_*i*_^l^] to numerically solve eqs 15 and 16 within the range of 0 ≤ *z* ≤ *L*.

In our calculations, we choose *L* = 50σ_1_ and *Δz* = 0.02σ_1_. Here σ_1_ is the diameter of a polyol segment. Our choice for *L* is 8–10 times larger than the width of the interface. This allows us to accurately resolve the spatial density profiles at the interface and the bulk. The equations are solved using Picard iteration with a convergence criterion that the deviation of the Euclidean distance between two consecutive density profiles is <10^–6^. Because we solve cDFT equations in an open system, the location of the vapor–liquid interface is translationally invariant. Hence, we fix the spatial position of the interface. At the beginning of the iteration process, the position of the interface is at *z* = *L*/2 with the following spatial density profile:
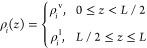
After every 10th iteration step, the entire profiles are shifted left or right, so that the Gibbs dividing surface^69^ relative to the polyol is located at *L*/2.

### 2.3 Incipient Phase Calculation to Initiate Bubble Nucleation

The primary objective of this work is to investigate the effect of the SPE surfactant on the nucleation of a spherical CO_2_ bubble in polyol. To initiate bubble nucleation, we first saturate the polyol–surfactant mixture with CO_2_ at the desired high pressure and temperature (303.8 K). We label this as the saturated state. The initial high pressure dictates CO_2_ solubility in the saturated state. The desired CO_2_ weight fraction in a foam formulation is ∼0.2–0.3 (w/w), and this is realized at pressures of 6–7 MPa. For reference, the critical point in the CO_2_ phase diagram is at 7.38 MPa and 303.8 K. Then, we instantly decrease the pressure to ambient conditions keeping the temperature and the CO_2_ weight fraction in the system fixed. This leads to a metastable state in which CO_2_ is supersaturated in the system. We refer to this state as the metastable parent phase.

We solve the PC-SAFT EoS at 1 atm pressure and 303.8 K to determine the densities of different components in the metastable parent phase, while keeping the CO_2_ content unchanged from that in the saturated state. The metastable parent phase serves as a starting point for the nucleation of a CO_2_-rich bubble.

Because nucleation is a rare event, the system undergoes local density fluctuations representative of the microstates that are visited during the formation and breaking of subcritical nuclei. In our quasi-thermodynamic approach to nucleation, we seek to identify the CO_2_-rich bubble that is in chemical potential equilibrium with the metastable parent phase. We hypothesize that such a CO_2_-rich bubble is the incipient phase that the metastable parent phase tends to form; the composition of the incipient phase represents that of a large, well-formed bubble. We note that the pressure inside the incipient CO_2_-rich bubble is greater than the ambient pressure (i.e., the pressure of the metastable parent phase). As a result, the nucleated bubble eventually expands. In this work, we focus on the MFEP to the incipient CO_2_-rich bubble from a metastable parent phase and the surfactant effect on the associated free energy barriers. We solve cDFT equations with the string method^45,70^ in spherical coordinates. At each point along the string, the system is at a constrained equilibrium, which allows the free energy of the bubble to be calculated. Connecting the points along the string results in the MFEP for the nucleation of an incipient CO_2_-rich spherical bubble.

### 2.4 String Method for the MFEP of Bubble Nucleation

Within a mean-field framework, the MFEP for the nucleation is the most likely path that connects the initial and final metastable states via a transition state.^45,70^ On a hypersurface characterized by the grand potential (Ω[{ρ}]), let {ρ_*i*_(**r**, *s*)|0 ≤ *s* ≤ 1} be a smooth curve (called a string) connecting the initial state {ρ_*i*_(**r**, *s* = 0)} (metastable parent phase, homogeneous polyol–surfactant mixture supersaturated with CO_2_) and the final state {ρ_*i*_(**r**, *s* = 1)} (incipient vapor-like spherical bubble). Here, *s* is the normalized reaction coordinate along the path. Then the string method attempts to determine the MFEP by evolving this smooth curve such that the tangent along the path is parallel to the free energy gradient.
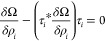
17where,
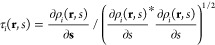
is the normalized tangent along the path and the asterisk denotes the inner product [i.e., f*g = ∫*f*(**r**)*g*(**r**) d**r**]. If we have only the first term in eq 17, i.e., , then the equation is the same as the standard free energy minimization problem. Solutions to such an equation would correspond to either the metastable parent phase (corresponding to *s* = 0) or the final spherical bubble phase (*s* = 1). Adding the second term to eq 17, i.e.,, facilitates the exploration of the intermediate states along the path connecting *s* = 0 and *s* = 1.

We solve eq 17 using a modified steepest descent algorithm to enforce the particular parametrization of the string.^48^ The iteration starts with a set of initial density profiles between the initial state (*s* = 0) and the terminal state (*s* = 1, a well-developed bubble). States between *s* = 0 and *s* = 1 are obtained by linear interpolation. After each iteration, we reparametrize the states of the density profile equidistantly along the path. The process ends when the relative difference in the free energy along the path between two consecutive iterations is <10^–5^. As noted by Müller and co-workers,^71,72^ such a steepest descent approach to constructing the string in the MFEP does not account for the dynamic constraint of local mass conservation. However, local extrema including saddle points in the free energy are unaffected by the local mass conservation. Therefore, we believe the qualitative picture for nucleation presented here remains valid.

### 2.5 Model Parameters and System Composition

The pure component parameters for describing CO_2_ and polyol come from the work of Xu et al.^47^ and Ylitalo et al.^73^ The corresponding parameters for describing different segments in the silicone polyether (SPE) surfactant are determined through a group contribution method.^74,75^ These parameters are listed in Table 1. For a nonpolar or weakly polar system, such as the ternary system of interest to this work, the binary interaction correction term *k*_*ij*_ that is used to correct for the dispersion interactions is negligibly small and is expected to have a minimal effect on the relevant properties of the system. Hence, in our modeling of ternary systems, we set the binary interaction correction terms to zero (i.e., *k*_*ij*_ = 0).

**Table 1:** PC-SAFT Parameters for Describing a CO_2_–Polyol–Surfactant Ternary System^a^

segment type	σ (Å)	ϵ/*k*_B_ (K)	*m*
CO_2_	2.79	170.5	2
PDMS	3.46	204.58	3.58
PPO	2.99	226.5	2.57

a For segments of type PDMS and PPO, *m* represents the number of segments per monomer. For CO_2_, *m* represents the number of segments per molecule.

For our study, we work with a model linear polyol with a molecular weight of 2700 g/mol. We choose to investigate the properties of different silicone surfactants whose overall molecular weights are close to that of the polyol. In this context, it is worth noting that a pure PDMS chain with a molecular weight as low as 1112 g/mol (equivalent to 40 PC-SAFT PDMS segments here) almost completely phase separates out of polyol. The presence of PPO type segments in the silicone surfactant is expected to improve the solubility of the silicone surfactant in polyol and render it surface-active. To systematically characterize the behavior of silicone surfactant in the CO_2_–polyol–silicone surfactant ternary system, we consider surfactants with different fractions of PDMS and PPO per chain. We acknowledge that the surfactant architecture affects their shape, aggregation, and interfacial properties.^76^ However, in this work, we consider the simpler case of a PDMS_*Nf*_-PPO_*N*(1–*f*)_ type linear diblock surfactant, where *N* is the total number of segments per chain and *f* is the fraction of PDMS segments per chain. SPE surfactants that are commonly used in foam formulations have *f* values of ∼0.3–0.6.^27^ Hence, we restricted our analysis to surfactants with *f* values of ≤0.42. The different surfactants studied in this work are all listed in Table 2. Such a choice of surfactants helps us to systematically investigate the trends in the aggregation behavior of a silicone surfactant in polyol, the effect of the CO_2_ on it, and the vapor–liquid interfacial tension of the CO_2_–polyol–silicone surfactant ternary system. The results from these calculations are reported and discussed in the following section.

**Table 2:** Chemical Compositions of Different SPE Surfactants Studied in This Work

surfactant	molecular weight (g/mol)	fraction of PDMS segments per chain (*f*)	PDMS weight fraction per chain
PDMS_30_-PPO_90_	2859.0	0.25	0.29
PDMS_35_-PPO_85_	2885.0	0.29	0.34
PDMS_40_-PPO_80_	2912.0	0.33	0.38
PDMS_50_-PPO_70_	2965.0	0.42	0.47

## 3 Results and Discussion

### 3.1 Micellization of a SPE Surfactant in Polyol

The critical micelle concentration (CMC) is a characteristic property of a surfactant. It is the surfactant concentration in a solution above which most surfactant molecules exist in the form of aggregates. Micelles are reported to constrain the rate of drainage of the liquid from the film between bubbles, leading to a stable foam.^20^ Micelles may also serve as seeds for gas adsorption, thereby promoting bubble formation through the heterogeneous nucleation pathway. Hence, the knowledge of the CMC of an SPE surfactant in polyol is crucial for the design of stable foams.

At any given surfactant concentration, micellar aggregates of different sizes form and break apart in the system. Their formation is always energetically favorable. However, when the surfactant concentration is lower than its CMC, the formation of these micelles (relative to the homogeneous bulk solution) is unfavorable due to the translational entropy loss of the individual surfactant molecules. If the surfactant concentration is higher than its CMC, micelles will form in large numbers. This serves as a working definition for determining the CMC of a surfactant.^77^

**Figure 1:**
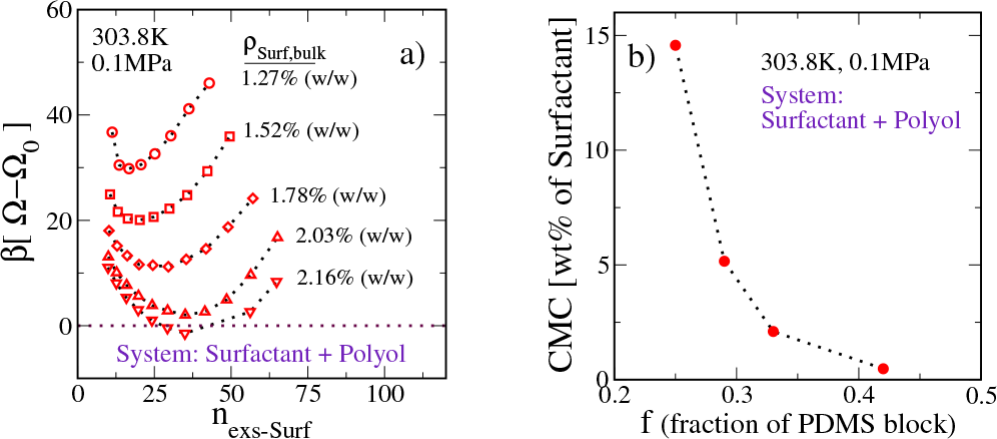
Micellization of a PDMS_*Nf*_-PPO_*N*(1–*f*)_ type SPE surfactant in polyol. (a) Micelle formation free energy [β(Ω – Ω_0_)] as a function of excess surfactant (*n*_exs–surf_) in the system. Here, *N* = 120 and *f* = 0.33. The CMC is defined as bulk surfactant concentration ρ_surf,bulk_ for which the most probable micelle has zero formation free energy. (b) CMC of SPE surfactants with *N* = 120 and different fractions (*f*) of PDMS block per chain.

In Figure 1a, we report the formation free energy of a micelle as a function of its size for different surfactant concentrations in the bulk solution. We define the micelle formation free energy (*βΔΩ*_mf_) as β(Ω – Ω_bulk_), where *βΩ* is the grand potential of the system containing the micelle and *βΩ*_bulk_ is that of a bulk solution. A micellar aggregate of the desired size is obtained by restraining the polyol density,^78^ to half its bulk value, at a given radial distance from the micelle center. To determine the size of such a micelle, we define the following order parameter: *n*_exs–surf_ = ∫d**r**[ρ_surf_(**r**) – ρ_surf,blk_]. This order parameter measures the excess number of surfactant molecules in the system relative to the homogeneous bulk solution. We see that, at any given surfactant concentration, the micelle formation free energy has a characteristic minimum. This free energy corresponds to the micellar aggregate with the most probable size. An increase in the surfactant concentration leads to a decrease in the formation free energy of the most probable micellar aggregate. At a suitable composition, the formation free energy of the most probable micellar aggregate attains a value equal to zero. We identify this as the surfactant’s CMC. Beyond the CMC, the formation free energy of the most probable micellar aggregate becomes negative. Thus, it is thermodynamically favorable to produce micelles in large numbers. For the PDMS_40_-PPO_80_ surfactant in polyol shown in Figure 1, the CMC is found to be ∼2.16% (w/w) surfactant in the polyol–surfactant mixture. For reference, the amount of surfactant that is commonly used in a foam formulation is ∼1–5% (w/w).

A surfactant’s tendency to aggregate into micelles can be enhanced by increasing the number of unfavorable interactions between the surfactant and the polyol. In this context, a key contributing parameter is the fraction (*f*) of the PDMS segments of the surfactant. In Figure 1b, we report the micellization of different PDMS_*Nf*_-PPO_*N*(1–*f*)_ type SPE surfactants in polyol. We fix *N* to 120 segments and vary *f* to study the effect of the length of the solvophobic block on surfactant micellization. We see that an increase in the fraction of PDMS segments from 0.25 to 0.42 leads to a decrease in the CMC from ∼15% (w/w) to ∼0.1% (w/w). With more solvophobic PDMS groups, the surfactant is less soluble in polyol. As a consequence, the surfactant tends to aggregate at lower concentrations.

### 3.2 Effect of CO_2_ on the Micellization of the SPE Surfactant in Polyol

**Figure 2:**
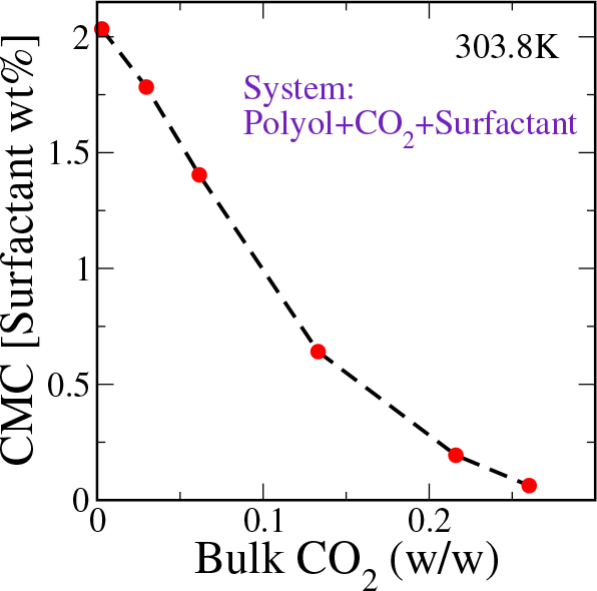
CMC of a PDMS_40_-PPO_80_ type SPE surfactant at different concentrations of dissolved CO_2_ in polyol.

In a foaming system, bubble nucleation is initiated by supersaturating the polyol–surfactant mixture with CO_2_. To investigate the effect of the surfactant on CO_2_ bubble nucleation in polyol, it is then necessary to understand surfactant micellization as a function of the amount of dissolved CO_2_ in the system. As noted in Models and Methods, we control the CO_2_ content by the system pressure. The higher the system pressure, the more CO_2_ is dissolved. In Figure 2, we report trends in the CMC of an SPE surfactant as a function of CO_2_ content in the CO_2_–polyol–SPE surfactant solution.

When there is no CO_2_, the CMC of a model PDMS_40_-PPO_80_ type SPE surfactant is found to be ∼2% (w/w) surfactant in the solution. An increase in the CO_2_ content leads to a decrease in the CMC. When the CO_2_ composition is ∼0.2% (w/w), the CMC is found to be as low as 0.1% (w/w) surfactant in the solution.

**Figure 3:**
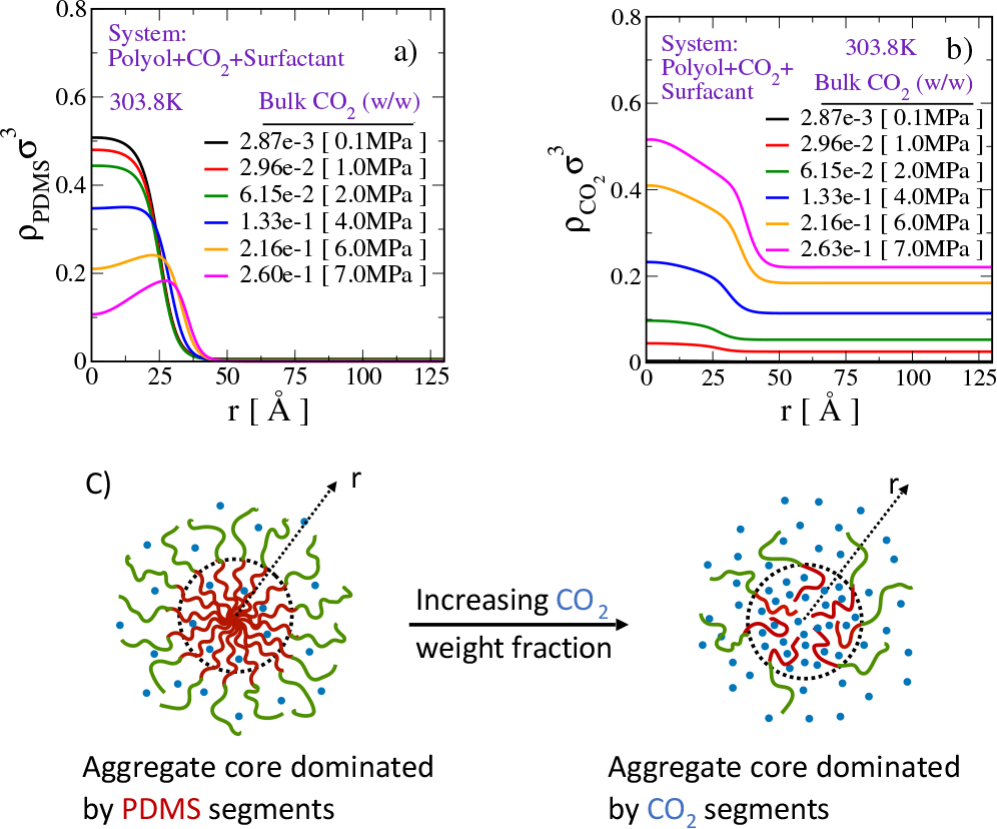
Spatial density profiles of (a) PDMS and (b) CO_2_ segments in a CO_2_-saturated polyol mixture at the CMC of the PDMS_40_-PPO_80_ type SPE surfactant in the system. Here, *r* represents distance from the center of the micellar core and σ is the unit of length. Values in the square brackets in the legends represent the pressure (in megapascals) at which the desired CO_2_ saturation in the system is attained. (c) Cartoon illustration of the transition from a PDMS (red curly lines)-dominated micellar core to the CO_2_ (blue dots)-dominated core with increase in the degree of CO_2_ saturation in polyol. The green curly lines in the cartoon represent the PPO block of the SPE surfactant.

To understand the effect of CO_2_ on the surfactant’s CMC, we examine the spatial density profiles of PDMS and CO_2_ segments in the most probable micellar aggregate (at the surfactant’s CMC). We see from Figure 3a that the interface is located at roughly the same spatial position, irrespective of the CO_2_ content of the solution. This suggests that all of these micelles have very similar micellar core sizes. However, an increase in the CO_2_ solubility in the solution decreases the density of the PDMS segments in the micellar core. As shown in Figure 3b, this decrease in the density of PDMS segments in the micellar core is compensated by a significant increase in the CO_2_ density in the micellar core.

When a sufficiently large amount of CO_2_ is dissolved in the mixture, the core of the micellar aggregate transforms from PDMS-dominated to CO_2_-dominated. Such a transformation is observed when the CO_2_ concentration in the bulk solution is ≥0.2% (w/w). A cartoon representing the PDMS-dominated and CO_2_-dominated micellar aggregates is depicted in Figure 3c.

The preferential partitioning of CO_2_ into the micellar core is a result of favorable CO_2_–PDMS interaction over CO_2_–polyol interaction. This results in swelling of the micellar core. However, we note that the micelle size is largely determined by the length of the PDMS block in the surfactant chain. As the micellar core is swollen due to the presence of CO_2_, the system now requires fewer surfactants to attain the same most probable micellar size. The presence of CO_2_ in the micellar core may also relieve the stress due to the dense packing of the PDMS segments therein. This coupled effect due to the CO_2_ may increase the drive for surfactants to aggregate more readily even at low surfactant concentrations.

### 3.3 Effect of the SPE Surfactant on the Vapor–Liquid Interfacial Tension in CO_2_–Polyol Mixtures

**Figure 4:**
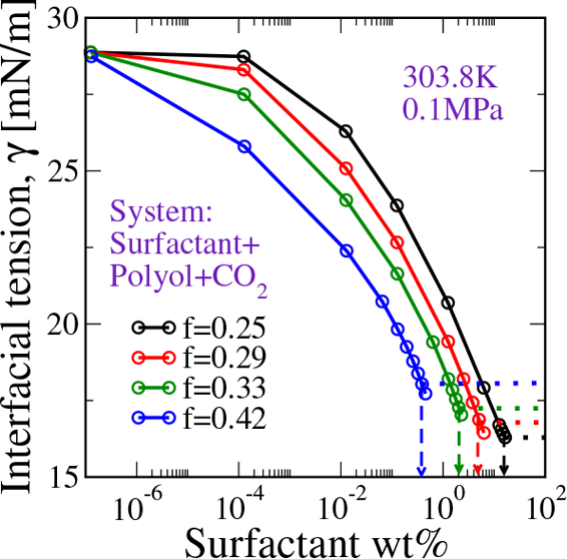
Interfacial tension between CO_2_-rich vapor and polyol-rich liquid at different concentrations of a PDMS_*Nf*_-PPO_*N*(1–*f*)_ type SPE surfactant in the system. Here, *N* = 120. Dashed lines with an arrow point to the CMC of the SPE surfacant in the system, and dotted lines represent the corresponding terminal interfacial tension, i.e., the interfacial tension at the surfactant CMC. Note that the portion of the curve below the dotted line for each *f* corresponds to surfactant concentrations that exceed the CMC and should be discounted, because the calculation of the interfacial tension does not directly account for micelle formation.

A polymeric foam is characterized by the presence of an interface between the CO_2_-rich gas and dense polymer-rich medium. A surfactant’s ability to reduce this interfacial tension facilitates the formation of such an interface. Here we investigate the interfacial tension between the CO_2_-rich vapor and polyol-rich liquid for the different surfactants studied in this work. The results of these calculations are summarized in Figure 4.

We find that, at a given weight percent of a PDMS_*fN*_-PPO_(1–*f*)*N*_ type SPE surfactant in the solution, increasing the fraction of the PDMS segments in the surfactant leads to a steep decrease in the interfacial tension. This is a consequence of the lower CMC for the surfactants with a higher fraction of PDMS segments per chain. Intriguingly, the terminal interfacial tension, i.e., the interfacial tension at the CMC, is found to be nearly insensitive to the PDMS content per surfactant chain. This suggests that all of these surfactants have very similar surface-active abilities at their respective CMCs. However, for the same total surfactant weight percent in the solution, a surfactant with a longer block of PDMS is more effective at reducing the interfacial tension. It is also worth noting that these surfactants are moderate in their abilities to reduce interfacial tension, with an only 10–15 mN/m decrease in the interfacial tension before attaining the CMC. Very similar observations were recorded from the experimental investigations by Kendrick et al.^19^

A surfactant’s ability to reduce the interfacial tension between the CO_2_-rich vapor and the polyol-rich liquid informs us only of the reduced penalty to form such an interface. However, to make predictions about the rate at which bubbles are generated, we need to understand the effects of the surfactant on the bubble nucleation pathway and the associated free energy barriers.

In modeling SPE surfactant-mediated CO_2_ bubble nucleation in polyol, we may consider nucleation when the surfactant’s concentration in the solution is (a) below its CMC and (b) at or above its CMC. In the former scenario, one is likely to find polyol, CO_2_, and surfactant to be uniformly dispersed in the solution. When the system is brought into the metastable state by a sudden decrease in pressure, such a system is likely to undergo homogeneous nucleation. In the latter scenario though, the presence of preformed micellar aggregates presents complex heterogeneous pathways toward bubble generation. For example, (1) micelles may serve as seeds for bubbles to nucleate, (2) micelles themselves may evolve into bubbles, or (3) micelles may disintegrate into smaller aggregates and then evolve into bubbles. In principle, our models can be extended to investigate each of these pathways. As a first step toward understanding surfactant-mediated CO_2_ bubble nucleation, we investigate only scenario (a) in this study, i.e., homogeneous nucleation.

### 3.4 Effect of the SPE Surfactant on the Homogeneous Nucleation of CO_2_ Bubbles in Polyol

We show that the addition of the surfactant not only reduces the barrier for CO_2_ bubble nucleation in polyol but also opens a new lower-energy barrier pathway through a spherical aggregate with a liquid-like CO_2_ core.

**Figure 5:**
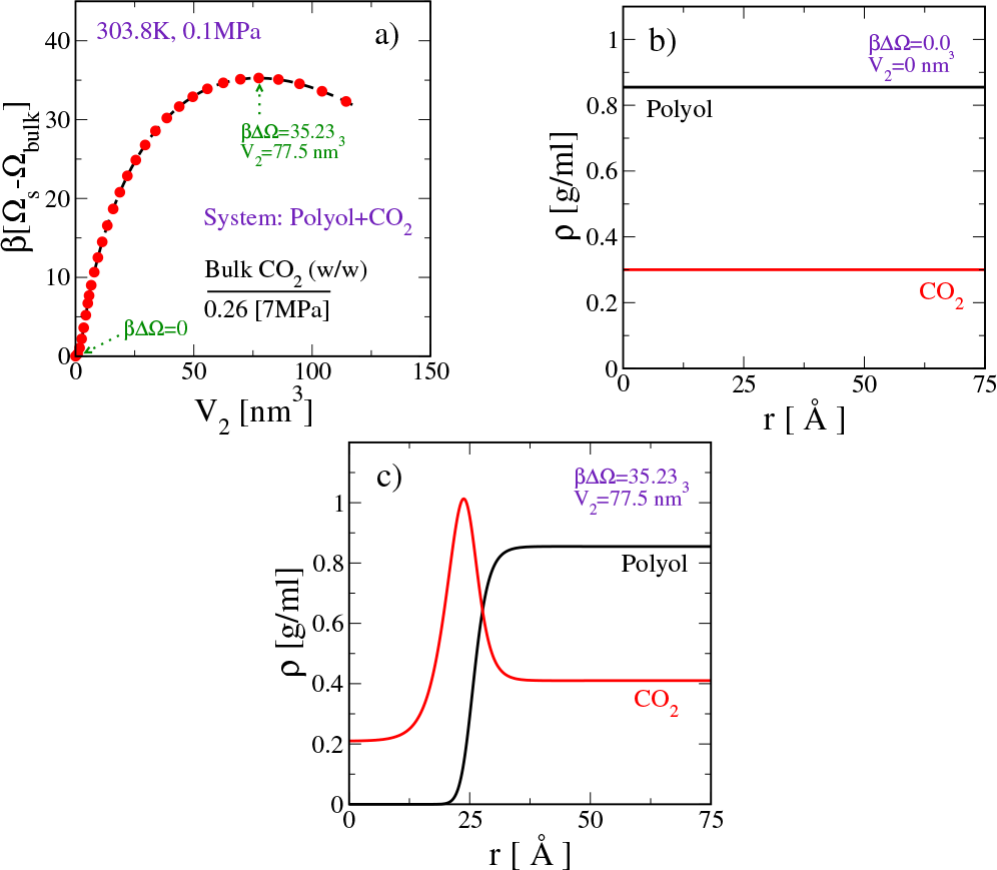
CO_2_ bubble nucleation in polyol when there is no surfactant in the system. (a) Minimum free energy path connecting the “homogeneous bulk solution” and a “spherical bubble” of predetermined size. *V*_2_ is the order parameter directly related to the size of the spherical bubble. Spatial density profiles of different components in (b) the “homogeneous bulk system” (*βΔΩ* = 0) and (c) the system at the “critical nucleus” (*βΔΩ* = 35.23). Here, *r* is the radial distance from the center of the spherical bubble and *βΔΩ* = β(Ω_s_ – Ω_bulk_), where Ω_s_ is the grand potential of the system with the bubble and Ω_bulk_ is that of the homogeneous bulk solution.

In Figure 5a, we report the MFEP for CO_2_ bubble nucleation in polyol when there is no surfactant. Here, the vertical axis represents the formation free energy of the bubble (relative to the homogeneous solution). The horizontal axis is an order parameter (*V*_2_) that quantifies the size of the bubble. The definition for *V*_2_ is
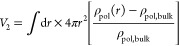
18

We see that the MFEP between the homogeneous solution and a vapor-like CO_2_ bubble passes through a single maximum in the free energy (see Figure 5a). At this maximum, the critical nucleus is CO_2_-rich and vapor-like, as seen in the density profiles in Figure 5c. Relative to the homogeneous solution (see Figure 5b), the free energy barrier corresponding to the critical nucleus is ∼35*k*_B_*T*.

**Figure 6:**
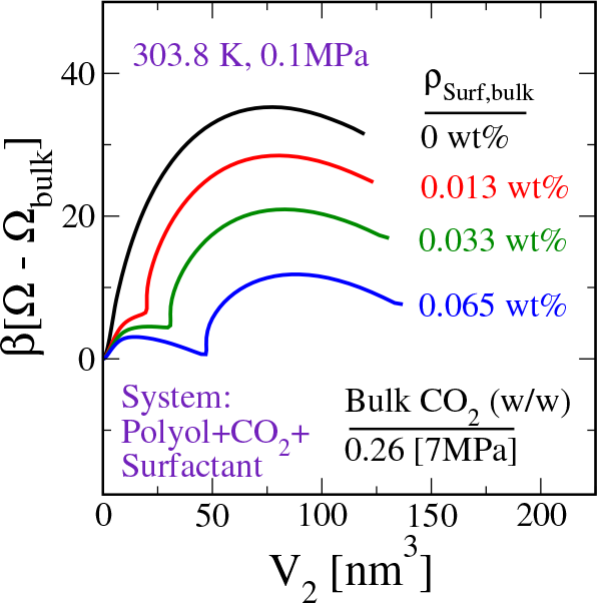
Minimum free energy path connecting the “homogeneous bulk solution” and a “spherical bubble of prescribed size” at different concentrations (ρ_Surf,bulk_) of a PDMS_40_-PPO_80_ type SPE surfactant in the system. Here, *V*_2_ is the order parameter directly related to the size of the spherical bubble. For reference, the CMC of this SPE surfactant in CO_2_–polyol mixture is ∼0.1% (w/w).

Adding the surfactant leads to significant changes in the MFEP for bubble nucleation. The results of these studies are reported in Figure 6. First, the formation free energy of the critical nucleus decreases with the surfactant concentration. Second, a shoulder appears during the early stages of the nucleation. This shoulder develops into a free energy barrier with an increase in the surfactant concentration.

**Figure 7:**
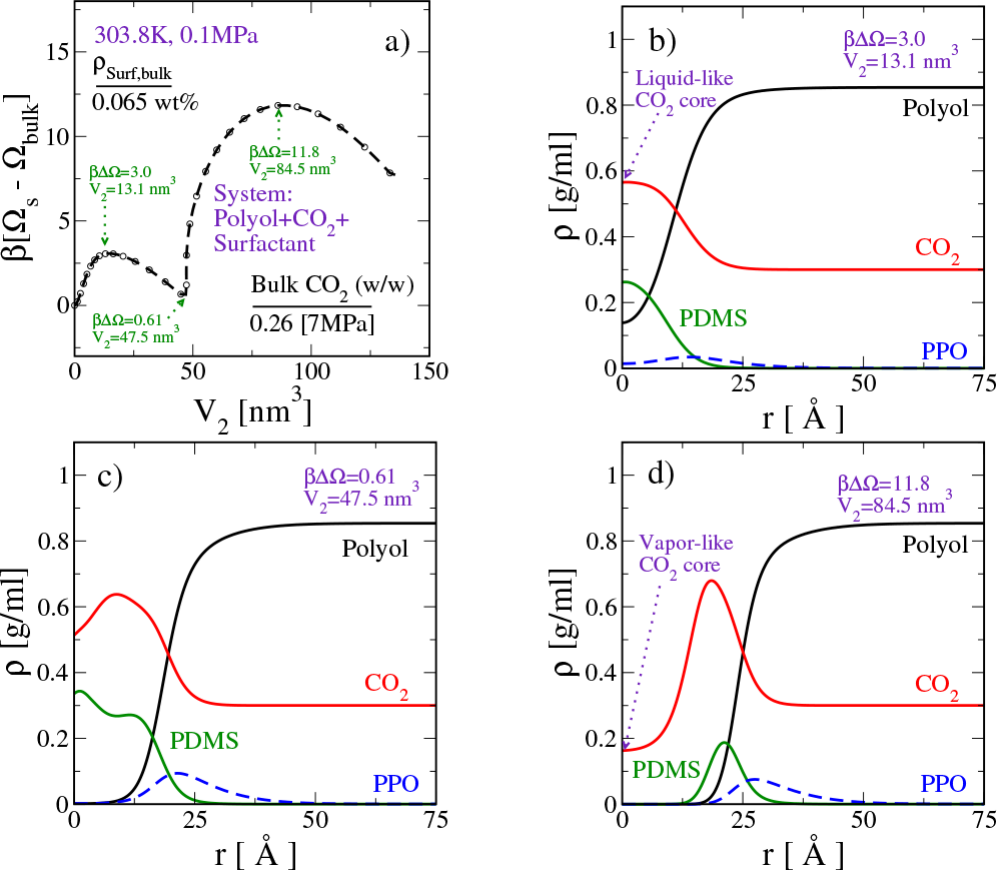
CO_2_ bubble nucleation in polyol upon the addition of a PDMS_40_-PPO_80_ type SPE surfactant to the system. (a) Minimum free energy path connecting the “homogeneous bulk solution” and a “spherical bubble of predetermined size”. *V*_2_ is the order parameter directly related to the size of the spherical bubble. (b–d) Spatial density profiles of system components at different stages along the minimum free energy pathway.

To understand the importance of the free energy barrier that appears during the early stages of the nucleation, we choose the system with a ρ_surf,bulk_ of 0.065% (w/w) as the model system and analyze the spatial density profiles along different stages of the nucleation. The relevant data are reported in Figure 7.

Figure 7a shows that the first barrier has a formation free energy of ∼3*k*_B_*T* and is lower than the value of ∼11.8*k*_B_*T* that is noted for the second barrier. From the density profiles in Figure 7b, we note that the spherical aggregate representing the latter is characterized by a ρ_CO_2_(*r*=0)_ of ≈0.6 g/mL. Due to the high density of CO_2_ in the core, we label this aggregate as the one with the liquid-like CO_2_ core. Formation of such an aggregate during the early stages of the nucleation and subsequent stabilization of the aggregate with a formation free energy of 0.61*k*_B_*T* (Figure 7c) may be a consequence of the favorable interactions of CO_2_–PDMS segments versus those of CO_2_–PPO segments. These aggregates eventually surpass an ∼11.0*k*_B_*T* barrier to vaporize into a spherical bubble with a vapor-like CO_2_ core.

The lower nucleation energy barrier resulting from the opening of a two-stage nucleation pathway upon the addition of surfactant could yield higher nucleation rates compared to that of a surfactant free solution. Such a pathway could yield foams with more, smaller bubbles, which can improve their microstructural features and insulating abilities.

## 4 Conclusion

In this work, using cDFT, we investigated the effect of a silicone polyether surfactant on CO_2_ bubble nucleation in polyol. We used a PC-SAFT EoS to model the chemically specific free energy functional for the cDFT calculations. Using these models, we first studied the interfacial and aggregation behavior of SPE surfactants in the CO_2_–polyol mixture. Following that, we computed the MFEP for CO_2_ bubble nucleation in polyol and discussed the effect of an SPE surfactant on such an MFEP.

We find that the SPE surfactants aggregate into micelles in the CO_2_–polyol mixture. The terminal air–liquid interfacial tension of such a system is found to be 10–15 mN/m lower than that of the system without the surfactant. The CMC of an SPE surfactant is found to be strongly dependent on the CO_2_ content of the system. The CMC decreases with increase in the CO_2_ system. In a typical foam formulation, the surfactant’s composition is ∼1–5% (w/w) and CO_2_ is saturated to ∼25% (w/w). Our calculations suggest that the surfactant’s CMC is ∼0.1% (w/w) when the CO_2_ content is ∼25% (w/w), implying that the surfactants may have aggregated into micelles even before the system is brought into the CO_2_-supersaturated metastable state. Similar to nanoparticles that impact foam production,^79,80^ the presence of micelles may promote bubble nucleation, alter foam microstructure, and enhance foam stability. Though modeling heterogeneous nucleation is beyond the scope of this work, models similar to those presented in this work have been employed in the past to explore related problems like nanoparticle solvation in a polymer–CO_2_ mixture.^81^ In the future, we plan to extend the current model to study CO_2_ bubble nucleation in the presence of preformed micelles.

When the surfactant concentration in the CO_2_–polyol mixture is below its CMC, it is likely that the CO_2_ bubble nucleates from a homogeneous solution of the CO_2_–polyol–surfactant mixture. In such a case, we find that the surfactant reduces the free energy barrier for CO_2_ bubble nucleation in polyol. While the free energy barrier is ∼30*k*_B_*T* when there is no surfactant, it is found to be as low as 10*k*_B_*T* upon addition of 0.05% (w/w) SPE surfactant. Interestingly, the associated MFEP changes from a single-step nucleation process to a two-step nucleation process in the presence of a surfactant. From the density profiles, we find that the first barrier corresponds to the formation of a spherical aggregate with the liquid-like CO_2_ core while the second barrier represents an aggregate with a vapor-like CO_2_ core. We hypothesize that the formation of a spherical aggregate with a liquid-like CO_2_ core during the early stages of bubble nucleation leads to a lower-energy barrier path for CO_2_ bubble nucleation in polyol. This can enhance the nucleation rate and ultimately result in the production of foams with a reduced pore size and an increased number density of pores.

In this work, we focused on only the bubble nucleation aspects during the foam production process. However, for a comprehensive understanding, it is equally critical to investigate how surfactants constrain bubble coarsening and bubble coalescence. Stierle and Gross have recently reported a dynamic density functional theory (DDFT) to study bubble coalescence.^82^ In their DDFT approach, the authors have accounted for the viscous forces as well as diffusive molecular transport through generalized Maxwell–Stefan diffusion. Similar to our work, Stierle and Gross use a PC-SAFT EoS to model the free energy functional for their DDFT. Suitable extension of their model to the SPE surfactant–CO_2_–polyol system would address the effects of surfactants on stabilizing polymer foams by constraining bubble coarsening and coalescence.
